# Antigen B from *Echinococcus granulosus* enters mammalian cells by endocytic pathways

**DOI:** 10.1371/journal.pntd.0006473

**Published:** 2018-05-04

**Authors:** Edileuza Danieli da Silva, Martin Cancela, Karina Mariante Monteiro, Henrique Bunselmeyer Ferreira, Arnaldo Zaha

**Affiliations:** 1 Programa de Pós-Graduação em Biologia Celular e Molecular, Centro de Biotecnologia, Universidade Federal do Rio Grande do Sul, Porto Alegre, Rio Grande do Sul, Brazil; 2 Laboratório de Biologia Molecular de Cestódeos, Centro de Biotecnologia, Universidade Federal do Rio Grande do Sul, Porto Alegre, Rio Grande do Sul, Brazil; 3 Laboratório de Genômica Estrutural e Funcional, Centro de Biotecnologia, Universidade Federal do Rio Grande do Sul, Porto Alegre, Rio Grande do Sul, Brazil; The First Affiliated Hospital of Xinjiang Medical University, CHINA

## Abstract

**Background:**

Cystic hydatid disease is a zoonosis caused by the larval stage (hydatid) of *Echinococcus granulosus* (Cestoda, Taeniidae). The hydatid develops in the viscera of intermediate host as a unilocular structure filled by the hydatid fluid, which contains parasitic excretory/secretory products. The lipoprotein Antigen B (AgB) is the major component of *E*. *granulosus* metacestode hydatid fluid. Functionally, AgB has been implicated in immunomodulation and lipid transport. However, the mechanisms underlying AgB functions are not completely known.

**Methodology/Principal findings:**

In this study, we investigated AgB interactions with different mammalian cell types and the pathways involved in its internalization. AgB uptake was observed in four different cell lines, NIH-3T3, A549, J774 and RH. Inhibition of caveolae/raft-mediated endocytosis causes about 50 and 69% decrease in AgB internalization by RH and A549 cells, respectively. Interestingly, AgB colocalized with the raft endocytic marker, but also showed a partial colocalization with the clathrin endocytic marker. Finally, AgB colocalized with an endolysosomal tracker, providing evidence for a possible AgB destination after endocytosis.

**Conclusions/Significance:**

The results indicate that caveolae/raft-mediated endocytosis is the main route to AgB internalization, and that a clathrin-mediated entry may also occur at a lower frequency. A possible fate for AgB after endocytosis seems to be the endolysosomal system. Cellular internalization and further access to subcellular compartments could be a requirement for AgB functions as a lipid carrier and/or immunomodulatory molecule, contributing to create a more permissive microenvironment to metacestode development and survival.

## Author summary

Antigen B (AgB) is an oligomeric lipoprotein highly abundant in *Echinococcus granulosus* hydatid fluid. AgB has already been characterized as an immunomodulatory protein, capable of inducing a permissive immune response to parasite development. Also, an important role in lipid acquisition is attributed to AgB, because it has been found associated to different classes of host lipids. However, the mechanisms of interaction employed by AgB to perform its functions remain undetermined. In this study, we demonstrate that mammalian cells are able to internalize *E*. *granulosus* AgB in culture and found that specific mechanisms of endocytosis are involved. Our results extend the understanding of AgB biological role indicating cellular internalization as a mechanism of interaction, which in turn, may represent a target to intervention.

## Introduction

Cystic hydatid disease (CHD), caused by the larval stage (hydatid or metacestode) of parasites belonging to the *Echinococcus granulosus sensu lato* (s.l.) complex, is a zoonosis of worldwide occurrence, with a considerable medical and economic impact [1]. CHD is endemic or hyperendemic in South America, especially in Argentina, Southern Brazil, Uruguay, Chile and mountainous regions of Peru and Bolivia [2]. In 2010, the World Health Organization added CHD to its list of Neglected Tropical Diseases (http://www.who.int/neglected_diseases/diseases/en/). *Echinococcus granulosus sensu stricto*, or simply *Echinococcus granulosus*, is one of the cryptic species of the *E*. *granulosus* s.l. complex and is the species most widely distributed worldwide. Also, *E*. *granulosus* is responsible for most cases of human CHD infections [3].

The adult tapeworm lives in the small intestine of a definitive canid host, and the larval stage develops in the viscera of a wide range of mammal species, including humans. *E*. *granulosus* life cycle is predominantly domestic, where dogs are the definitive hosts and ungulates are the intermediate hosts [4]. The metacestode is a fluid-filled, unilocular cyst that is classified as fertile when protoescoleces are present in the lumen. Protoescoleces are the pre-adults, infective to the definitive host, which remain quiescent and immersed in the hydatid fluid (HF), which is a complex mixture of molecules of both host and parasite origin. The excretory/secretory products of the metacestode are of special relevance for the host-parasite relationship, as they have a greater potential to interact with host proteins and cells.

Antigen B (AgB) is a lipoprotein, highly abundant and the major immunodominant protein among the excretory/secretory metacestode products in *E*. *granulosus* HF. AgB belongs to the group of hydrophobic ligand binding proteins (HLBPs), a cestode protein family whose members are known by their high abundance and immunogenicity, and by their oligomeric structure, comprising 7–10 kDa α-helix rich subunits [5–7]. AgB oligomers have been observed predominantly in the molecular mass range of 150–230 kDa, but aggregates with higher molecular masses have also been detected [8,9]. The AgB oligomeric structure comprises 8 kDa subunits (AgB8/1 to AgB8/5) encoded by a multigene family [10], which are differentially expressed among *E*. *granulosus* life-cycle stages, metacestode tissues and individuals [8,11,12]. The subunits AgB8/1 to AgB8/4 have been identified compounding the proteic moiety of *E*. *granulosus* native AgB from bovine and human HF samples. AgB8/1 was the most represented subunit in both bovine and human samples. AgB8/3 had distinct relative abundancy between the different hosts, it was the less abundant in bovine samples and it was found in relative high abundance in human samples [8]. A recent study with AgB particles obtained from fertile HF of swine origin, infected by *Echinococcus canadensis*, a closely related specie, also found AgB8/1 as the most represented subunit. Additionally, the authors found AgB8/4, AgB8/3 and AgB8/5, while AgB8/2 was not detected [13].

It has been demonstrated that delipidated AgB is able to bind hydrophobic compounds *in vitro* [14]. The lipid moiety associated with AgB represent 40–50% of the total particle mass and is constituted by different lipid classes, from hydrophobic lipids, like sterol esters and triacylglycerides, to a variety of phospholipids [9,13]. Moreover, delipidated recombinant AgB8/2 and AgB8/3 subunits were capable of transferring fatty acids analogues to artificial phospholipid membranes [15]. *E*. *granulosus* genome lacks sequences for several key enzymes for fatty acid and cholesterol synthesis, thus the parasite is incapable of synthesizing these compounds *de novo* [16,17]. Hydatid viability relies on the sequestration and utilization of host lipids, and AgB might be involved in lipid uptake from host tissue and its transport to the parasite, by stabilizing insoluble lipids into a lipoproteic particle [9].

Helminths are said to elicit a modified type 2 response, which consists in Th2 profile with an anti-inflammatory component, probably involving T_reg_ stimulation [18]. AgB roles in the modulation of both innate and adaptive immunity have been proposed. It has been described that neutrophils have both the recruitment inhibited and hydrogen peroxide production decreased by AgB [19,20]. Immature dendritic cells stimulated with AgB matured and primed lymphocytes towards the type 2 response [21]. Also, a Th2 polarization in the Th1/Th2 cytokine ratios was observed in AgB-stimulated peripheral blood mononuclear cells from patients with active CHD [22].

Considering the two main roles attributed to AgB, immunomodulation and lipid transport, it is reasonable to consider that a direct interaction with host cells and tissues should occur. In fact, it was recently demonstrated that AgB binds to macrophages and the plasma membrane of inflammatory monocytes, inducing a non-inflammatory phenotype in macrophages [23]. However, little is known about the molecular details of AgB-cell interaction and whether AgB interacts with non-immune cells, or even enters into the cell.

In the present work, we investigated the ability of HF-purified AgB to enter into different mammalian cell types *in vitro*, and the mechanisms involved in AgB internalization. Immunopurified AgB was incubated with four distinct cell lines representative of different cell types, namely hepatocytes, fibroblasts, macrophages, and lung epithelial cells. We demonstrated the entry of AgB into the cytoplasm of all studied cell lines. Moreover, we provided evidence that the endocytic pathways are involved in AgB internalization by cells, with caveolae/raft-mediated endocytosis being the prevailing one.

## Methods

### Biological material

Hydatids were obtained from bovine viscera of naturally infected animals slaughtered in the routine work of a local abattoir (São Leopoldo, Brazil). Animal slaughtering was conducted according to Brazilian laws and under supervision of the *Serviço de Inspeção Federal* (Brazilian Sanitary Authority) of the Brazilian *Ministério da Agricultura*, *Pecuária e Abastecimento*. *Echinococcus* spp contaminated viscera, identified during mandatory meat inspection, were donated by the abattoir for use in this work. HF was removed by punction and aspiration from individual fertile cysts and kept at -80°C until use. Protoescoleces were collected for parasite genotyping and species determination [24]. Only cysts belonging to *E*. *granulosus* (G1 strain) were used in this work.

### Immunoblot

Aliquots of 100 μl of individual *E*. *granulosus* HF samples were resolved on SDS-PAGE 12% and electrophoretically transferred onto a nitrocellulose membrane. A pool of rabbit polyclonal antibodies raised against each recombinant AgB subunit (AgB8/1 to 5) were used at 1:70.000 dilution as primary antibody. The pool of antibodies corresponds to the IgG fraction of each subunit specific serum purified using HiTrap ProteinG HP columns (GE Healthcare), according to the manufacturer's protocol. A horseradish peroxidase-conjugated goat anti-rabbit IgG (GE Healthcare) diluted at 1:7.000 was used as the secondary antibody. Blots were developed using the chemiluminescent reagent ECL Plus (Pierce, ThermoScientific) and imaged in VersaDoc system (BioRad). HF samples with higher AgB content were used for the protein purification step (S1A Fig).

### *E*. *granulosus* AgB purification

AgB purification was carried out following the protocol described by Oriol *et al*. [25], with some modifications. Briefly, parasite proteins from individual *E*. *granulosus* HF were precipitated by sodium acetate (5 mM, pH 5.0) and the resultant material was resuspended in phosphate-buffered saline (PBS) containing 20 μM 3,5-di-tert-butyl–4-hydroxytoluene (BHT). The HF parasite enriched fraction, obtained from individual cysts, was subjected to immunoaffinity chromatography using primary IgG antibodies against the recombinant forms of AgB8/1, AgB8/2 and AgB8/4 (see “Immunoblot” section). Antibodies were separately coupled to cyanogen bromide-activated Sepharose 4B resin (GE Healthcare) and the previously prepared HF material was passed through the columns. Bound AgB from each column was eluted with 100 mM tris-glycine pH 2.5, pooled together to reconstitute the original HF sample, then dialyzed against PBS/BHT and concentrated on Amicon Ultra-15 centrifugal filter device, MWCO 3 kDa (Millipore). Resultant purified AgB from individual cysts was kept separated and analyzed on SDS-PAGE 12% (S1B Fig). AgB concentration was determined using a Qubit quantitation fluorometer and Quant-iT reagents (Life Technologies).

### Blue Native PAGE (BN-PAGE)

BN–PAGE was carried out using the NativePAGE Novex Bis–Tris Gel System (Invitrogen) according to the manufacturer’s specifications. Precast NativePAGE Novex 4–16% Bis–Tris gels were run at 150 V for 1.5–2 h in 50 mM BisTris/Tricine pH 6.8 running buffer, where 0.02% Coomassie G-250 was added to the cathode buffer. Protein samples (10 μg) were mixed with the sample buffer provided. Gels were stained also according to the NativePAGE Novex Bis–Tris Gel System instructions. Briefly, the gel was firstly placed in fix solution (40% methanol, 10% acetic acid), then in destain solution (8% acetic acid) until desired background was obtained.

### Cell cultures

A549 (human lung adenocarcinoma, European Collection of Authenticated Cell Cultures: ECACC 86012804), J774.A1 (mouse macrophages, *Banco de Células do Rio de Janeiro*: BCRJ code 0121), NIH-3T3 (mouse fibroblasts, American Type Culture Collection: ATCC CRL-1658) and RH (rat Reuber hepatoma, ATCC CRL-1600) cells were cultivated in DMEM containing 10% fetal bovine serum, 100 U/ml penicillin and 100 μg/ml streptomycin in a 5% CO_2_ humidified environment at 37°C. J774.A1 culture media was also supplemented with MEM non-essential amino acid solution, 2 mM glutamine, 10 mM HEPES and 1 mM sodium pyruvate. All cells lines were free from mycoplasma contamination.

### AgB internalization assays

NIH-3T3, A549, J774.A1 and RH cells were grown on sterile glass coverslips in 35 mm Petri dishes until 70–80% of confluency. Cell media was changed to serum-free medium and the cells were then incubated with 40 μg/ml of AgB for 4 h at 37°C, or 4°C. Controls were incubated with equal volume of PBS/BHT. Unbound protein was then removed by three washes with cold PBS and cells were fixed in 4% paraformaldehyde/PBS at room temperature for 15 min.

In all microscopy preparations, a pool of the same antibodies used for AgB purification (anti-AgB8/1, anti-AgB8/2 and anti-AgB8/4) was used as primary antibody for detection of AgB. Fixed cells were permeabilized with 0.2% Triton X-100/PBS and unspecific sites were blocked with 5% BSA in PBS-T (PBS with 0.05% Tween-20). After, cells were incubated overnight at 4°C with the primary antibodies (1:500) and then for 1 h with 1:200 diluted Alexa Fluor 488-conjugated anti-rabbit secondary antibody (Molecular Probes) at room temperature. Nuclei were stained with 100 nM 4',6-diamidino-2-phenylindole (DAPI) (Molecular Probes). Actin was stained with 50 nM Alexa Fluor 594-conjugated phalloidin (Molecular Probes). Cells were imaged using a LSM 710 Zeiss confocal microscope.

The fluorophore CM-DiI (Molecular Probes) was used to directly label AgB particle, because it has affinity to the lipidic compounds associated to the protein. DiI-labelled AgB was used to analyze internalization without cells fixation. AgB was labelled with 5 μM CM-DiI (Molecular Probes) for 1 h at room temperature. Dye excess was washing out with 5-fold the original PBS volume on Amicon Ultra-0.5 centrifugal filter devices, NMWL 100 kDa (Millipore). RH cells were incubated with 40 μg/ml of DiI-labelled AgB for 4 h at 37°C, washed three times with cold PBS, and immediately analyzed using an Olympus FluoView 1000 confocal microscope.

### Endocytosis inhibition assays

RH and A549 cell monolayers were grown until 70–80% of confluency on sterile glass coverslips in six-well tissue culture plates. After changing the cell media to serum-free DMEM, cells were pre-treated with endocytosis inhibitors for 30 min at 37°C. A pilot test, where cells were incubated with different concentrations of the inhibitors, was conducted to determine the best concentration to be used. The highest concentration where >80% of the cells remained attached and with little morphological alterations was chosen.

Genistein (Santa Cruz Biotechnology) was used at 100 μg/ml concentration and chlorpormazine (Santa Cruz Biotechnology) at 5 μg/ml. AgB was then added at 40 μg/ml and after incubation at 37°C for 1.5 h, the unbound proteins were removed by acidic stripping (0.5 M NaCl, 0.5% acetic acid, pH 3.0) and three washes with cold PBS. Cells were fixed and prepared for microscopy as described above. Cells were imaged using an Olympus FluoView 1000 confocal microscope. Immunofluorescence intensity normalized by cell area was assessed with ImageJ software [26]. Image analysis was done on two (A549) or three (RH) independent experiments, where three microscopy fields were counted for each experiment (100–300 cells/experiment).

### Colocalization assays

RH cell monolayers were grown until 70–80% of confluency on sterile glass coverslips in 6-well tissue culture plates. Cell media was replaced to serum-free DMEM containing 40 μg/ml AgB and the distribution of internalized protein was compared with that of different endocytic markers following up to 1.5 h incubation at 37°C. Endogenous transferrin receptors were labeled with 50 μg/ml Alexa Fluor 633-conjugated transferrin (Tfn) (Molecular Probes), added in the last 45 min. Alexa Fluor 555-conjugated cholera toxin subunit B (Ctx-B) (Molecular Probes) at 1 μg/ml concentration was added in the last 15 min of incubation. Adsorbed and unbound proteins were removed by acidic stripping (0.5 M NaCl, 0.5% acetic acid, pH 3.0) and three washes with cold PBS. Cells were prepared for microscopy and imaged as described for endocytosis assay.

To analyze colocalization of AgB with the endolysosomal system, RH cells were incubated with 40 μg/ml DiI-labelled AgB (described in “AgB internalization assays” section) and 1.5 μM Lysosensor Green DND-189 (Molecular Probes). After 1.5 h incubation at 37°C, cell monolayers were washed three times with cold PBS and immediately analyzed by confocal microscopy.

Colocalization of AgB with Ctx-B, Tfn or Lysosensor was assessed using JaCoP plugin from ImageJ software [27]. Image analysis was done for two independent experiments.

### MTT reduction assays

A549 and RH cells were plated onto 96-well plates at a density of 10^4^ cells/well. AgB were added to the cell media at 2.5–40 μg/ml final concentrations. After 24 h incubation, 0.5 mg/ml of MTT solution in PBS was added to each well and incubated for a further 4 h. To solubilize formazan, 100 μl of cell lysis buffer (16% SDS, 40% N,N-dimethylformamide, 2% acetic acid, pH 4.7) was added to each well and the samples were incubated overnight at 37°C in a humidified incubator. Absorbance values of formazan were determined at 595 nm with an automatic microplate reader (Bio-Rad, model 550). Analysis was done for five independent experiments.

### Statistics

A Kolmogorov-Smirnov was applied to verify the normality of the data. Statistical significance was analyzed by unpaired Student’s t-test using the GraphPad Prism 6.0 software. Data are expressed as mean ± SEM and p values of less than 0.05 were considered statistically significant.

## Results

### AgB is internalized by mammalian cells in culture

The ability of AgB particles to interact with and to be internalized by mammalian cells was investigated using AgB immunopurified from *E*. *granulosus* HF. The native AgB is able to form particles with variable masses, thus the AgB preparation is probably heterogeneous in size. To have some clue on the predominant particles present in our AgB samples, a BN-PAGE analysis was carried out. The presence of a major band corresponding to ~160 kDa, as well as, a band corresponding to higher molecular mass and a smear, which could be resultant of particles with variable molecular masses confirm that our AgB preparation fits the information on literature regarding the molecular mass expected for native AgB (S1C Fig).

**Fig 1:**
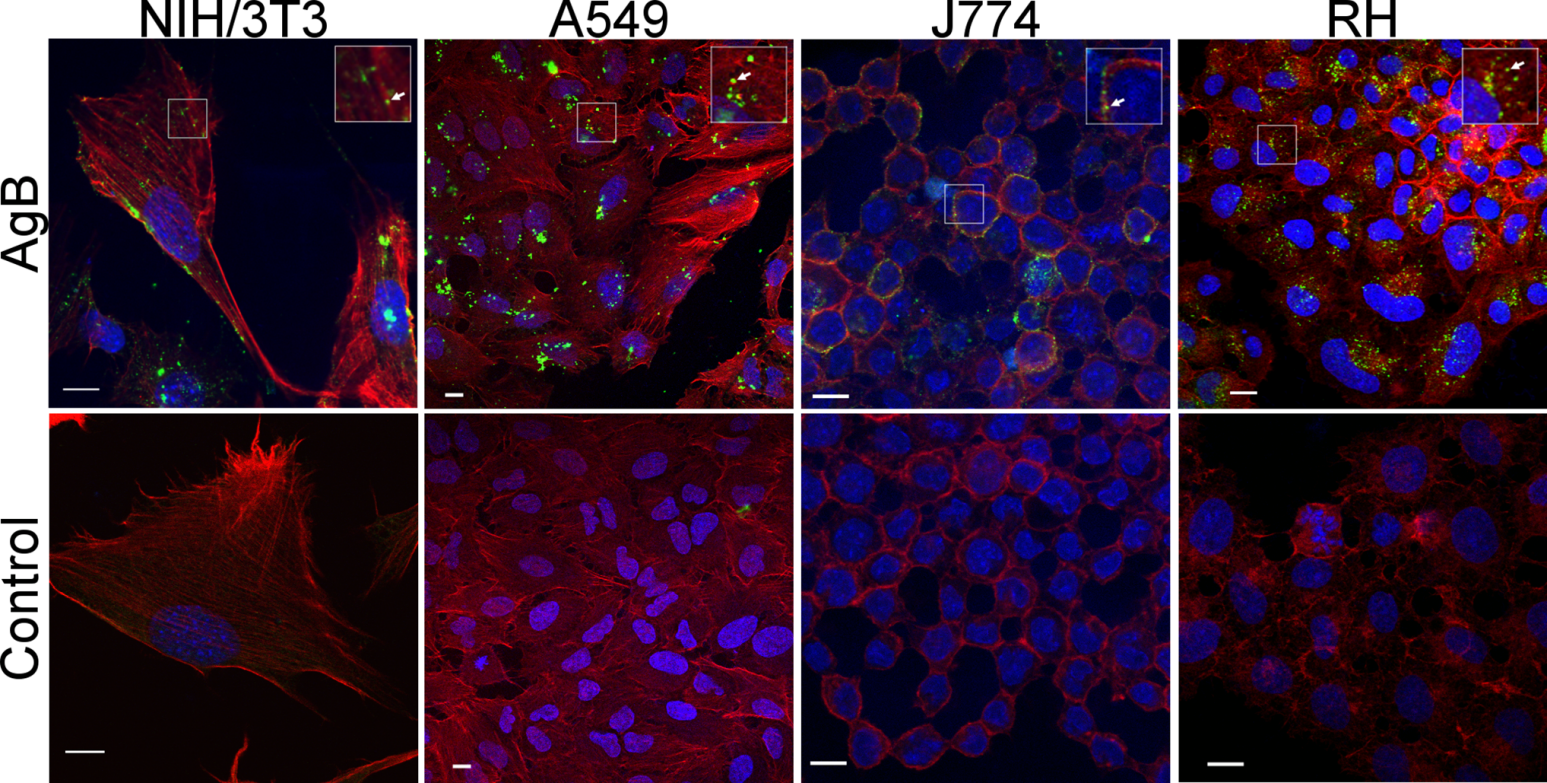
*E*. *granulosus* AgB uptake by mammalian cells in culture. Immunofluorescence assay was performed on NIH-3T3, A549, J774 and RH cells exposed to 40 μg/ml AgB for 4 h, and mock treated cells (Control). AgB was labeled with polyclonal antibodies against AgB8/1, 2 and 4 subunits and an Alexa Fluor 488-conjugated secondary antibody (green). Nuclei and cytoskeleton were stained with DAPI (blue) and Alexa Fluor 594-conjugated phalloidin (red), respectively. Images are median optical sections from z-stacks obtained by confocal microscopy. Inserts correspond to two-fold digital magnification of boxed areas. Arrows indicate vesicular-like distribution of internalized AgB. Scale bar, 10 μm.

Immunopurified AgB was added to the culture medium of NIH-3T3, A549, RH or J774 cells and internalization was evaluated after 4 h of incubation at 37°C using an immunofluorescence assay. Cells were prepared for confocal microscopy by labelling AgB with polyclonal antibodies against AgB8/1, 2 and 4 subunits and a secondary anti-rabbit IgG conjugated to Alexa Fluor 488. AgB signals were detected in the four cell lines tested, and no differences in extension of AgB internalization was observed, thus suggesting that AgB is able to interact with mammalian cells by a mechanism independent of cellular type. No signals were detected in the cells without AgB (Fig 1).

To confirm the internal localization of AgB in the cells, the lipoprotein was labelled with DiI and incubated with RH cells in the same way as before. However, the analysis on confocal microscope was conducted right after incubation had been finished, without cell fixation. The intermediate sections from confocal z-stacks showed higher AgB signal than top or bottom sections, confirming that AgB was inside cells and not just adsorbed to cell membrane (S2 Fig).

AgB was detected in the cell cytoplasm, but not in the nucleus. In addition, Fig 1 and S2 Fig show vesicular-like distribution of AgB in the cytoplasm of the cell lines analyzed, indicating an internalization through endocytosis. Supporting this idea, AgB internalization does not occur when RH cells were incubated at low temperature (S3 Fig), a condition known to interfere in endocytosis-dependent cellular internalization [28].

### Endocytic pathways involved in AgB internalization

Having established that AgB could access the cytoplasmic compartment of mammalian cells, we then investigated which endocytic pathway could be responsible for this AgB uptake by RH and A549 cells. These two cell lines were chosen to perform the following experiments in an attempt to simulate the natural situation, as liver and lungs are the primary organs infected by *E*. *granulosus*.

**Fig 2:**
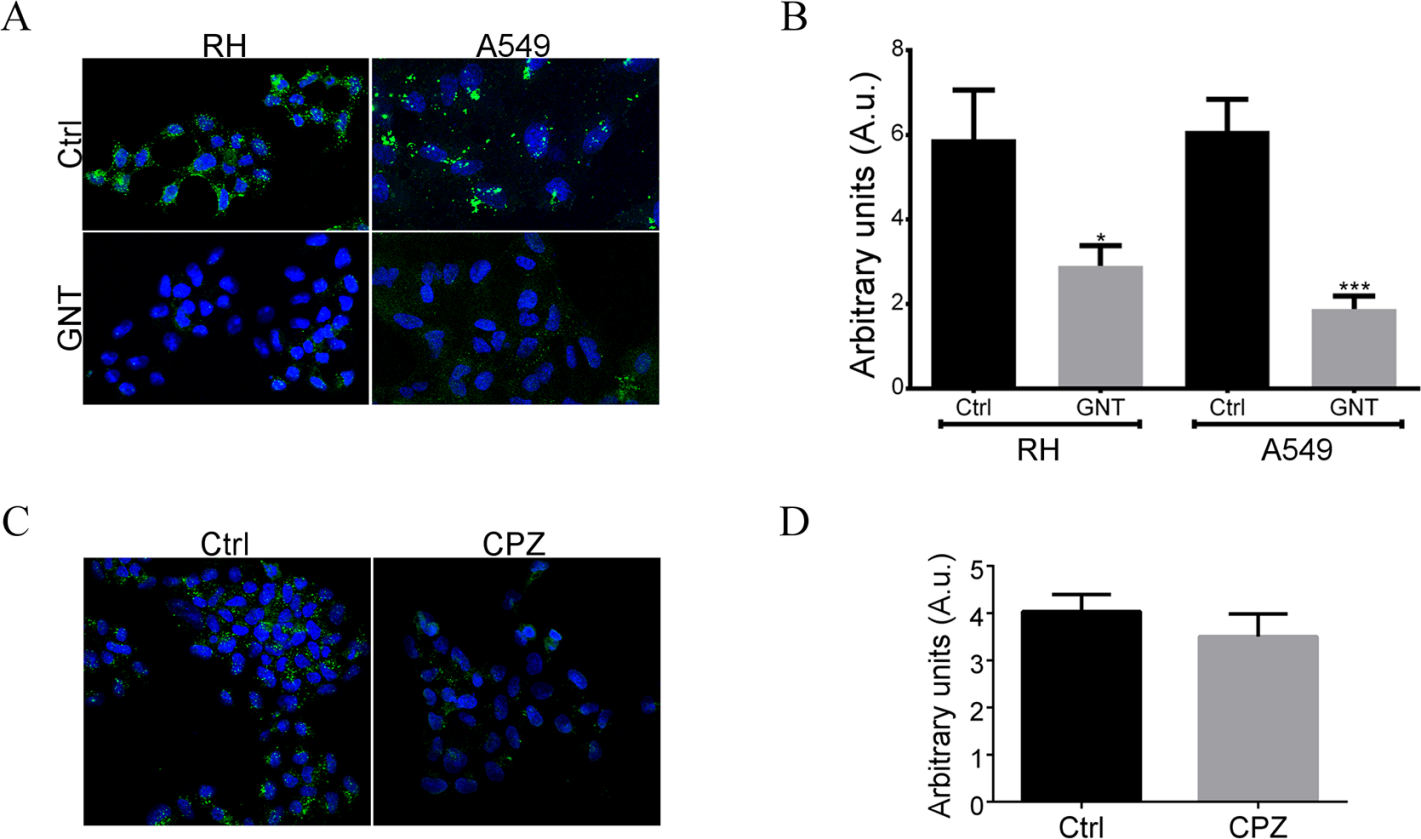
Caveolae/raft-mediated endocytosis is the main route involved in *E*. *granulosus* AgB internalization by A549 and RH cells. Indicated cells were incubated in medium alone (Ctrl), with 100 μg/ml genistein (GNT), or with 5 μg/ml chlorpromazine (CPZ) for 30 min and then exposed to 40 μg/ml AgB for 1.5 h. AgB was detected using anti-AgB polyclonal antibodies followed by anti-rabbit IgG Alexa Fluor 488 conjugated antibody (green). Cell nuclei were labelled with DAPI (blue). *A* and *B*, Inhibition of caveolae/raft-mediated endocytosis by genistein reduces AgB internalization. *C* and *D*, inhibition of clathrin-mediated endocytosis pathway by chlorpromazine does not cause a significant decrease in uptake of AgB. The quantitative data presented in B and D are measurements from three experiments with RH cells and two with A549 cells. Arbitrary units correspond to immunofluorescence intensity calculated as total immunofluorescence in the cell divided by the area of the cell. Error bars indicate SEM. *p = 0.037, ***p = 0.0004 according to Student’s t-test.

Genistein, a tyrosine kinase inhibitor that prevents caveoale/raft-mediated endocytosis, was used to treat RH cells; and after 30 min AgB were added to the culture media and left to incubate for another 1.5 h at 37°C. We found that internalization of AgB was inhibited by ~50% in RH cells treated with genistein (Fig 2A and 2B). The same inhibition assay was carried out with A549 cells and we found very similar results, where AgB uptake was inhibited by ~69% (Fig 2A and 2B). The results were statistically significant for both cell lines (Fig 2B).

In order to test whether other endocytic pathways could be involved in AgB uptake by RH cells, the inhibition assay was performed using chlorpromazine, an inhibitor of clathrin-mediated endocytosis. After chlorpromazine treatment, internalization of AgB was reduced by ~13%, however this was not statistically significant (p = 0.39) (Fig 2C and 2D). The higher inhibition of AgB internalization in the genistein treatment indicates that caveolae/raft-mediated endocytosis is the major pathway associated with AgB uptake.

The specificity of the inhibitors was confirmed using established endocytic markers as controls. Internalization of transferrin (Tfn), which undergoes clathrin-mediated endocytosis, was affected only by chlorpromazine. In contrast, genistein only reduced internalization of cholera toxin subunit B (Ctx-B), which undergoes caveolae/raft-mediated endocytosis (S4 Fig).

**Fig 3:**
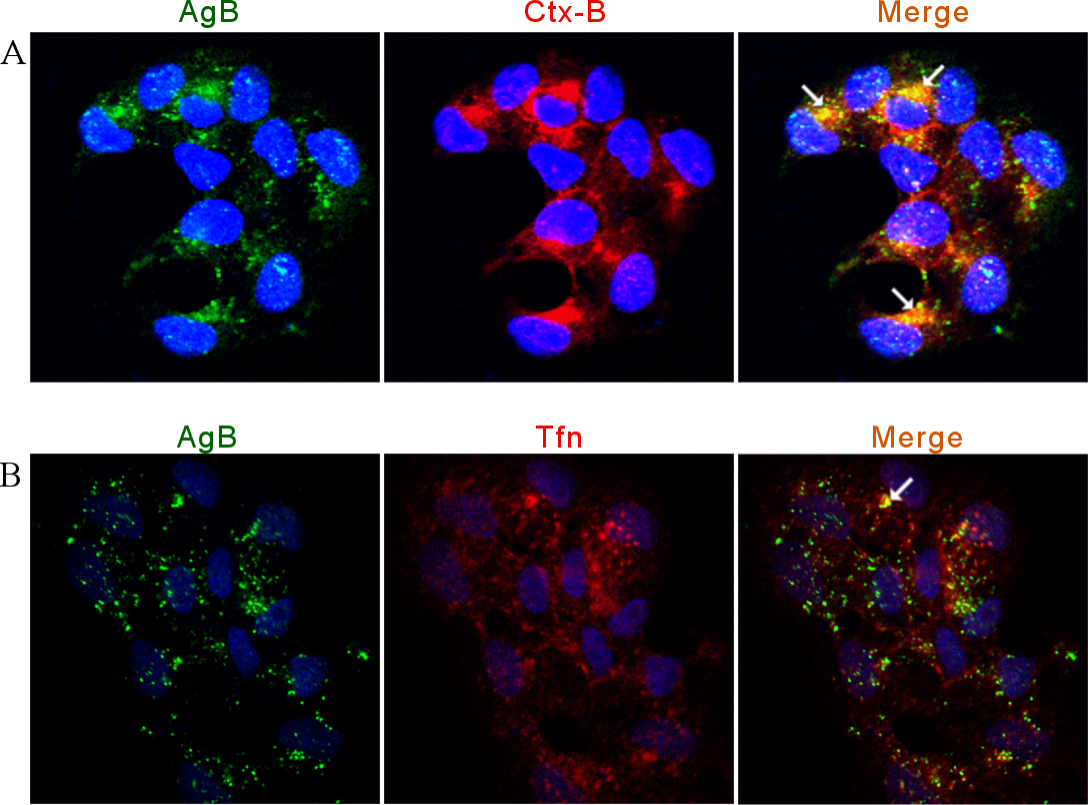
Internalized *E*. *granulosus* AgB colocalizes with protein endocytic markers in RH cells. Alexa Fluor 633-conjugated Tfn was used as a marker of clathrin-mediated endocytosis and Alexa Fluor 555-conjugated Ctx-B was used as a marker of caveolae/raft-mediated endocytosis. Confocal microscopy images of RH cells incubated with 40 μg/ml AgB and 1 μg/ml Ctx-B (A) or 50 μg/ml Tfn (B) are presented. AgB was detected using polyclonal antibodies against AgB8/1, 2 and 4 subunits and a secondary anti-rabbit IgG Alexa Fluor 488 conjugated antibody (green). Arrows indicate colocalization points. Cell nuclei were labeled with DAPI (blue). The endocytic markers are shown in red.

In a complementary approach to inhibition assays, the distribution of internalized AgB was compared with that of established endocytic markers, Tfn and Ctx-B. RH cells were cultivated in the presence of AgB for 1.5 h, with either Tfn or Ctx-B being added in the last 45 and 15 min of incubation, respectively. Results were analyzed by confocal microscopy and the level of colocalization between the two fluorophores and, consequently, the two proteins, was determined according to Pearson’s correlation coefficient. The results indicated that AgB was colocalized with Ctx-B (Pearson’s coefficient = 0.64 ± 0.02) (Fig 3), which is in accordance with our previous results that caveolae/raft-mediated endocytosis is involved in AgB uptake. Interestingly, AgB seemed partially colocalized with Tfn (Pearson’s coefficient = 0.49 ± 0.04), suggesting that in some degree AgB could be internalized by clathrin-mediated endocytosis.

Altogether, the above findings provide evidence that AgB entry into mammalian cells occurs mainly via caveolae/raft-mediated endocytosis, although it could also occur by clathrin-mediated endocytosis in a lesser extent.

### AgB is delivered to the endolysosomal system after internalization

**Fig 4:**
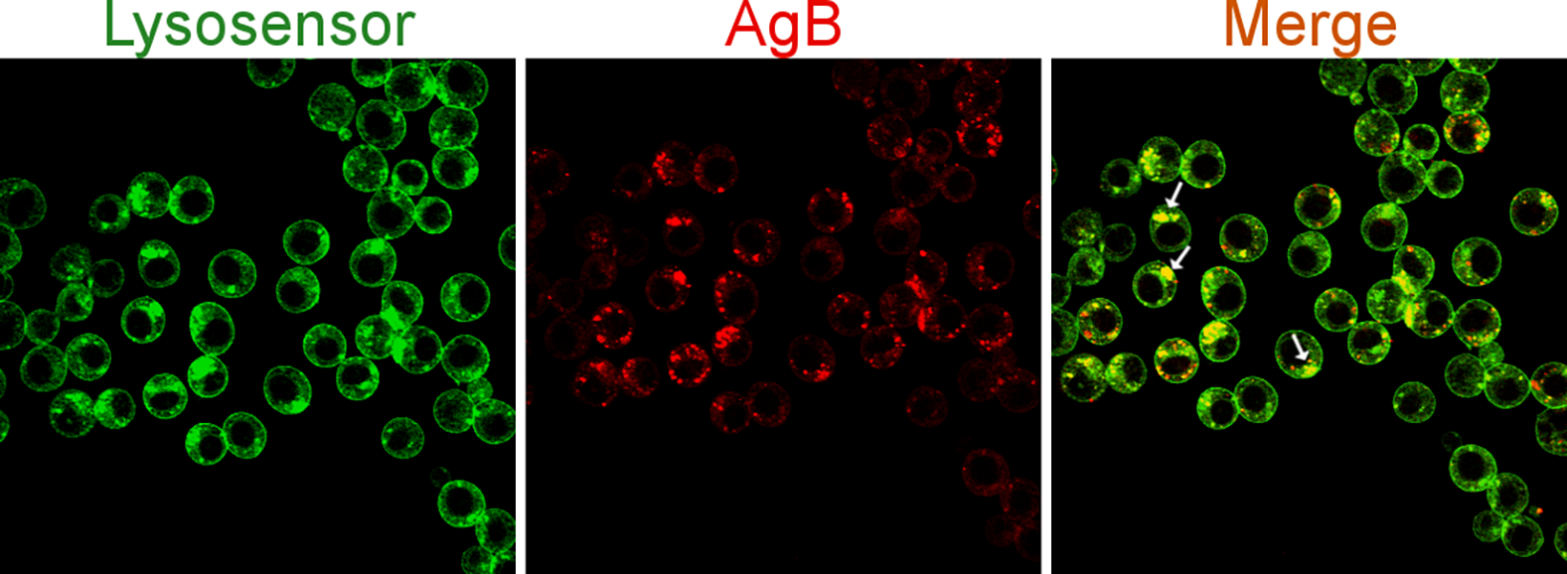
*E*. *granulsous* AgB reaches the endolysosomal system after endocytosis by RH cells. DiI-labelled AgB (red) at 40 μg/ml and Lysosensor DND-189 (green) at 1.5 μM were added to the cell culture media and left incubate for 1.5 h. Median sections from z-stacks of confocal images are shown. Arrows indicate colocalization points.

Lysosensor dyes are acidotropic probes that accumulate in acidic organelles in live cells and are used to track the endolysosomal system. To have some clue on AgB fate after endocytosis we sought to check whether AgB signal colocalized with late endosomes/lysosomes by using the Lysosensor DND-189 probe. Incubation of RH cells with DiI-labelled AgB and Lysosensor was carried out at 37°C for 1.5 h and colocalization was assessed in live cells using confocal microscopy. A considerable proportion of internalized AgB was found colocalizing with late endosomes/lysosomes (Pearson’s coefficient = 0.77 ± 0.008) (Fig 4). This result indicates that AgB trafficks in acidified compartments of endolysosomal system after uptake by cells.

### AgB does not induce cellular toxicity

**Fig 5:**
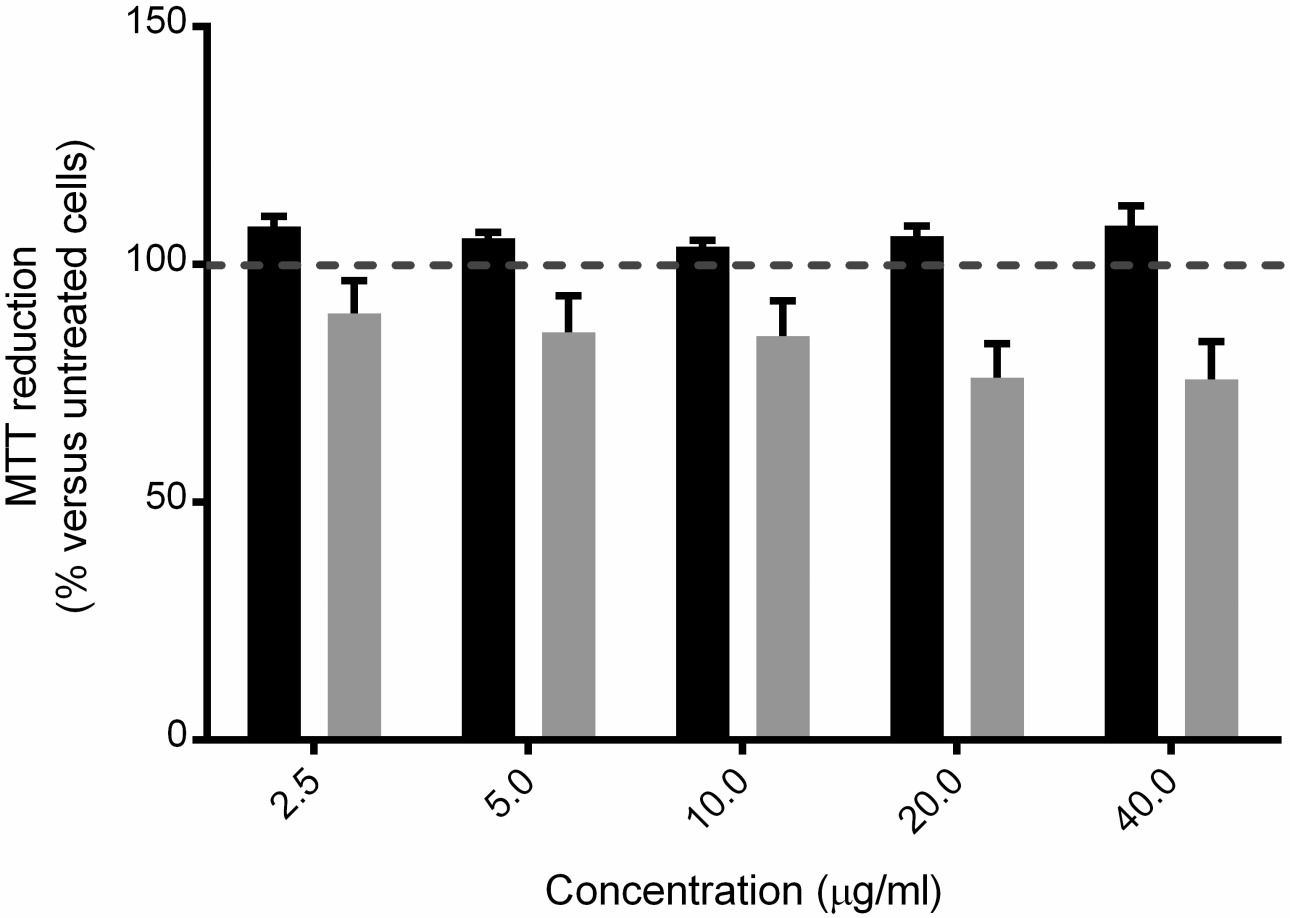
MTT reduction assay. A549 (black bars) and RH (gray bars) cells were treated for 24 h with the indicated concentrations of *E*. *granulosus* AgB. Cell viability is expressed as percentage of MTT reduction measured for untreated cells and assumed as 100% (horizontal dashed line). Error bars correspond to the SEM values of five independent experiments.

To further investigate the possible effects of AgB internalization by mammalian cells, A549 and RH cells were incubated with different concentrations of AgB (2.5–40 μg/ml) for 24 h. Alterations of cell physiological state were then evaluated by MTT reduction assays. Both cell lines did not present any significant decrease in their ability to metabolize MTT in presence of AgB (Fig 5). AgB do not have a cytotoxic effect on cells, so this interaction is probably part of the mechanisms underlying AgB function in host-parasite interplay.

## Discussion

A long-term growth is characteristic of the chronic infection caused by *E*. *granulosus* metacestodes, and to allow that, different molecular mechanisms are employed by the parasite to ensure its survival and development in the host microenvironment. In an attempt to evade the host’s immune response and absorb nutrients from the host, the parasite secretes several molecules. AgB is the major antigen in HF and has been implicated in immunomodulation processes, as well as in lipid uptake and transport [9,29].

AgB has been studied for a long time and it is known to generate a strong humoral response and to modulate host immune response, which is in agreement with the idea that AgB is secreted towards the outside of the hydatid cyst [29,30]. Moreover, there are many studies reporting AgB effects on immune cells that support a direct AgB-cell interaction in host tissue [21,23]. However, whether this interaction occurs with other cell types and what underlying mechanisms are involved in this process, are still unclear. We hypothesized that AgB may interact with host tissue cells surrounding the hydatid cyst to interfere with host cell homeostasis, facilitating nutrient acquisition and immune evasion. In this study, we demonstrated that *E*. *granulosus* AgB is taken up by mammalian cells *in vitro* by endocytosis. Since AgB internalization seems to be independent of the cell type, it most likely occurs by a ubiquitous mechanism. Thus, we further evaluate the involvement of specific endocytic pathways on AgB internalization.

Clathrin-mediated endocytosis is the best-understood internalization pathway and refers to intake of receptors and their bound ligands through vesicles which are coated by the protein clathrin [31]. Among the molecules known to be internalized by this pathway is the Tfn receptor, hence Tfn was used as a marker for clathrin-mediated endocytosis [32]. Besides the clathrin-mediated pathway, lipid rafts domains, and its subdomain caveolae, are important contributors to endocytosis processes. Caveolae/raft domains are heterogeneous membrane domains enriched in sphingolipids and cholesterol, and are involved in the endocytosis of various receptors and ligands with a multitude of mechanisms and regulation factors [33]. Ctx-B is a molecule that binds to glycosphingolipid GM1 on caveolae and non-caveolar rafts to be subsequently internalized, so we used it as a marker for caveolae/raft-mediated endocytosis [34]. Chlorpromazine and genistein were used in this study to inhibit clathrin- and caveolae/raft-mediated endocytosis pathways, respectively.

Treatment with genistein was able to significantly decrease AgB uptake by RH and A549 cells. Accordingly, we found that AgB colocalizes with Ctx-B in RH cells. We observed a partial colocalization of AgB with Tfn and the inhibition assay with chlorpromazine showed only a slight, not significant, decrease in AgB internalization. It is possible that clathrin-mediated endocytosis accounts for just a small part of AgB uptake, making difficult the detection of a difference after inhibition by chlorpromazine. Taken together, our results are consistent with the idea that the endocytosis process is required for AgB entry into mammalian cells. Indeed, caveoale/raft-mediated endocytosis is most likely the main pathway involved in AgB uptake by cells. However, a minor role for clathrin-mediated endocytosis in AgB internalization cannot be excluded.

It was proposed that AgB binds to macrophages and monocytes plasma membranes through a lipoprotein receptor; however no specific receptor could be determined [23]. As a lipoprotein, AgB may resemble the structure of some host lipoproteins, then tricking cell receptors to be internalized [9]. Our findings are in agreement with this idea because some lipoprotein receptors, such as lectin-like oxidized LDL receptor-1 (LOX-1), LDL receptor-related protein 6 (LRP6), and scavenger receptors CD36 and CD204 use caveolae/raft-mediated pathways for endocytosis [35–38]. Considering our results, it is also possible that more than one receptor might be involved in AgB binding, so that a higher efficiency of internalization is obtained. Alternatively, the receptor might undergo endocytosis by both pathways upon AgB binding. This regulatory mechanism involving different endocytic routes has been observed with LRP6, in which the receptor is internalized by caveolae to promote Wnt/β-catenin signaling transduction, whereas the clathrin route leads to LRP6 degradation [39].

In some molecules, structural and physicochemical features have central roles in interaction with membrane rafts. An amphipatic helix present in the tyrosine kinase interacting protein (Tip) from *Herpesvirus saimiri* has been shown target Tip to lipid rafts with cholesterol being important to the interaction [40]. Studies on nanoparticles (NP) uptake and dynamics have implicate surface characteristics on raft association and resultant NP mechanism of endocytosis. Lipidic NPs show higher affinity for caveolae as higher their lipophilicity [41], and NP probes designed with cationic surface are preferentially internalized by clathrin coated pits, while zwitterionic-lipophilic nanoprobes are preferentially internalized by lipid rafts [42]. AgB subunits are helix rich lipophilic proteins and predicted as amphiphilic molecules by *in silico* models [9,14,43]. These properties might promote AgB association with caveolaes/rafts lipidic components, which in turn could act as receptors and trigger the endocytosis process upon AgB binding.

AgB uptake did not induce toxicity to cells according to our MTT assay, therefore internalization is more likely part of the mechanisms underlying AgB roles during *E*. *granulosus* metacestode infection. *E*. *granulosus* genome lacks genes coding for several key enzymes involved in fatty acid and cholesterol synthesis [16,17]. On the other hand, *E*. *granulosus* AgB and other lipid binding proteins, like FABPs, are produced and secreted by the parasite to acquire lipidic compounds from host and supply parasite’s needs [44]. However, the mechanisms implicated in lipid capture by AgB are unknown. Since lipids are essential for metacestode survival and development, the understanding of molecular mechanisms employed by the parasites to acquire these host macromolecules will provide potential targets for therapeutic discovery efforts.

AgB interaction with cells from the host tissue surrounding the hydatid could be a suitable scenario to get lipids from biological membranes or inner cell storages. A similar scenario has been described for the *Taenia solium* metacestode, where HLBPs were able to translocate lipid analogs to parasite’s tissues, and also colocalize with lipid droplets in the granuloma surrounding the metacestode [6]. The mechanism of internalization of a lipoprotein to get lipids has been described in mammals too, where apolipoprotein A-I (apoA-I) binds macrophage (foam cells) and is internalized. The lipidated apoA-I particle is probably resecreted resulting in an efflux of cholesterol from the foam cell [45].

The oligomeric nature of AgB and its subunit composition could also have some relation with uptake by cells. Recombinant AgB subunits (AgB8/2 and AgB8/3), tested separately, were able to bind fatty acids analogs, but not cholesterol analogs. Cholesterol is found associated to the native particles, so AgB subunits could have selective capacity to bind lipids [15]. Similarly, AgB subunits could participate differently in the internalization process, for example, by varying in affinity for the ligand on the cell membrane. AgB particle is composed by a dozen subunits, but the actual proportion of each one into the final particle is unknown. It is possible that some subunits have major roles in lipid binding, while others are crucial to AgB-cell interaction. Future works testing the subunits individually are necessary to establish whether there is preference in cell association and internalization regarding each AgB subunit.

An endocytosed protein has many possible destinations inside a cell, including route to the trans-Golgi network, to the late endosomes and lysosomes for degradation or to recycling endosomes that bring part of cargo back to the plasma membrane [46]. A possible destination for AgB identified in this work was the acidic organelles in endolysosomal system, i.e., the late endosomes, the lysosomes and the product of their fusion, the endolysosomes. These components form the degradative branch of endocytic pathway, so as consequence of internalization, AgB could be degraded. However, there are intersects between the endolysosomal system and both the secretory pathway and retrograde transport to other organelles [34]. Multivesicular bodies (MVBs), for example, are derived from late endosomes and generate exosomes, that in turn, are released to extracellular milieu upon fusion of MVBs with the plasma membrane [47]. The MVBs could be a route of escape from degradation and redirection to secretion as exosome cargo [48]. However, we cannot exclude the possibility that the consequences of AgB presence in the endolysosomal system may be different according to the target cell. A more deep investigation is necessary to clarify if AgB goes to lysosome for degradation, or if it could escape and follow to secretory pathways with further exocytosis and return to parasitic tissues. Additionally, AgB-cell interaction may be a mechanism used by the parasite to create a more permissive microenvironment for metacestode development and survival. AgB presence in cytoplasm could interfere with cell metabolism, generating molecules and/or signals beneficial to the parasite.

Previous work showed AgB binding to macrophages induces a non-inflammatory phenotype, probably resultant of signaling through cell receptors [23]. In this work, we found that macrophages could also internalize AgB particles, expanding the possibilities on how AgB could modulate the immune response and influence the signalization towards non-inflammatory or alternative pathways. After endocytosis, AgB may access different cell compartments and interfere in cell processes related to immune activity as phagocytosis, antigen presentation, cytokine secretion, vesicles trafficking, etc. Similar mechanisms of immunoevasion were found in others helminth parasites. In *Fasciola hepatica* a cathelicidin-like protein was described to bind lipid rafts, and after internalization, to divert macrophages function by suppressing lysosomal activity and, consequently, interfering with antigen presentation [49]. Sm16 of *Schistosoma mansoni*, an oligomeric protein with membrane binding capacity, is quickly uptaken by macrophages, but after that is retained in early endosomes, delaying traffic to lysosomes and processing [50,51]. Retention in endosomes might mediate sequestration of essential TLR signaling machinery blocking stimulation of specific TLRs [51].

Like AgB, cestode HLBPs are involved in parasite lipid homeostasis and immunological process [6,52]. Thus, further investigations on the cellular and molecular effect of HLBPs on host cells are important steps to improve the understanding of the parasites biology and disease progression. It will be important to analyze AgB interaction with host-derived cells to confirm biological significance of caveolae/raft-mediated AgB endocytosis. Likewise, elucidating how the molecules sequestered by HLBPs become available to parasites cells will help to identify potential targets for the treatment and control of cestodiasis.

## Supporting information

S1 Fig *E*. *granulosus* AgB purification. (A) Immunoblot to confirm AgB presence in HF samples. An aliquot of 100 μl from each collected hydatid fluid was resolved on 12% SDS-PAGE and electrophoretically transferred onto a nitrocellulose membrane (lanes 1 to 8). A pool of anti-AgB subunit purified IgGs was used as the primary antibodies. (B) 12% SDS-PAGE analysis illustrating four independent AgB purifications, named 1 to 4. HF refers to hydatid fluid after precipitation by sodium acetate (5 mM, pH 5.0), resuspended in PBS containing 20 μM BHT. AgB refers to the samples eluted after immunoaffinity chromatography and concentrated to 1 ml final on Amicon Ultra-15 device. Aliquots of 10 μl were applied in the gel for both HF and AgB samples. The typical ladder-like pattern of native AgB is observed. Arrow indicates monomeric AgB. Arrowheads indicate AgB multimers. (C) BN-PAGE of immunopurified AgB. Samples (10 μg) from two independent purifications (1 and 2) were resolved on 4–20% BisTris/Tricine polyacrylamide gels and stained with Coomassie blue. The migration of molecular mass markers is indicated on the left of the gel. Protein markers were bovine thyroglobulin (669 kDa), bovine gamma-globulin (158 kDa) and bovine albumin (66 kDa).
(TIFF)

S2 Fig *E*. *granulosus* AgB uptake by RH cells. Cells were incubated with 40 μg/ml of DiI-labelled AgB for 4 h at 37°C. Images were acquired on a confocal microscope without cell fixation. Three different sections and orthogonal views (XZ and XY) for the intermediate section (middle panel) are shown. White lines indicate position of orthogonal views in XY plane. Scale bar, 10 μm.
(TIFF)

S3 Fig Endocytosis-mediated mechanisms are involved in AgB internalization. RH cells were exposed to AgB at 4°C for 4h, then fixed with paraformaldehyde. AgB was labelled with antibodies against subunits AgB8/1, 2 and 4 and an Alexa Fluor 488-conjugated secondary antibody (green). Nuclei and cytoskeleton were stained with DAPI (blue) and Alexa Fluor 594-conjugated phalloidin (red), respectively. Scale bar, 10 μm.
(TIFF)

S4 Fig Effects of genistein and chlorpromazine treatment on Ctx-B and Tfn internalization. RH cells were treated with 100 μg/ml genistein or 5 μg/ml chlorpromazine 30 min prior to addition of Alexa Fluor 555-conjugated Ctx-B or Alexa Fluor 633-conjugated Tfn. Cells were fixed in 4% paraformaldehyde and nuclei were stained with DAPI (blue). Ctx-B and Tfn are shown in red. Scale bar 10 μm.
(TIF)
