# Supplementary figures and images for: Antigen B from Echinococcus granulosus enters mammalian cells by endocytic pathways
Source: PLoS Negl Trop Dis. 2018 May 4;12(5):e0006473. doi: 10.1371/journal.pntd.0006473 (PMC5955594; doi:10.1371/journal.pntd.0006473)

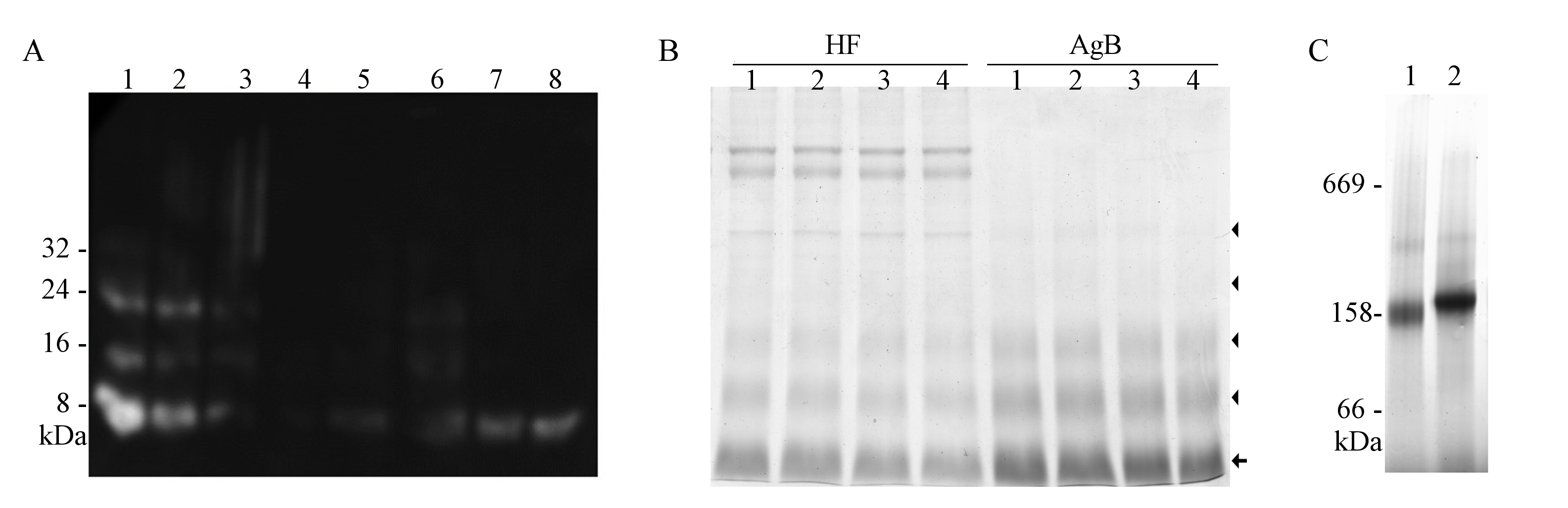

Supplement: S1 Fig — (A) Immunoblot to confirm AgB presence in HF samples. An aliquot of 100 μl from each collected hydatid fluid was resolved on 12% SDS-PAGE and electrophoretically transferred onto a nitrocellulose membrane (lanes 1 to 8). A pool of anti-AgB subunit purified IgGs was used as the primary antibodies. (B) 12% SDS-PAGE analysis illustrating four independent AgB purifications, named 1 to 4. HF refers to hydatid fluid after precipitation by sodium acetate (5 mM, pH 5.0), resuspended in PBS containing 20 μM BHT. AgB refers to the samples eluted after immunoaffinity chromatography and concentrated to 1 ml final on Amicon Ultra-15 device. Aliquots of 10 μl were applied in the gel for both HF and AgB samples. The typical ladder-like pattern of native AgB is observed. Arrow indicates monomeric AgB. Arrowheads indicate AgB multimers. (C) BN-PAGE of immunopurified AgB. Samples (10 μg) from two independent purifications (1 and 2) were resolved on 4–20% BisTris/Tricine polyacrylamide gels and stained with Coomassie blue. The migration of molecular mass markers is indicated on the left of the gel. Protein markers were bovine thyroglobulin (669 kDa), bovine gamma-globulin (158 kDa) and bovine albumin (66 kDa). (TIFF) [file pntd.0006473.s001.tiff]

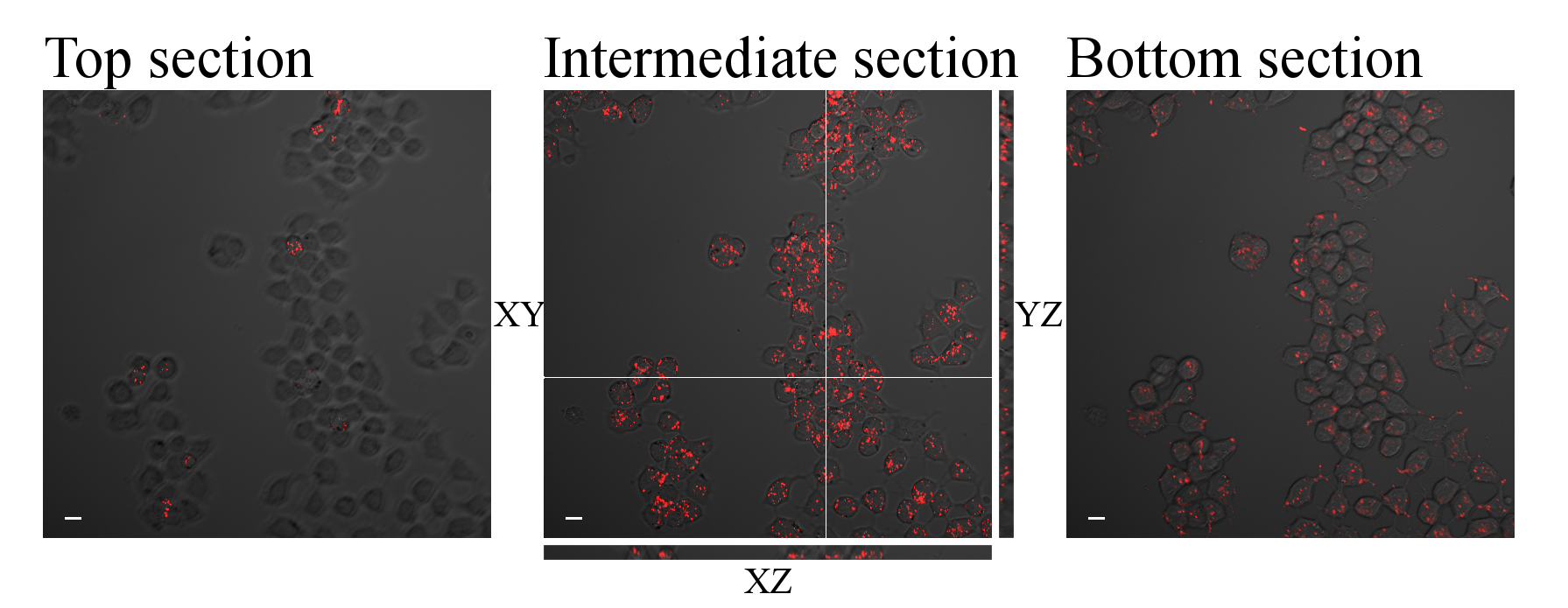

Supplement: S2 Fig — Cells were incubated with 40 μg/ml of DiI-labelled AgB for 4 h at 37°C. Images were acquired on a confocal microscope without cell fixation. Three different sections and orthogonal views (XZ and XY) for the intermediate section (middle panel) are shown. White lines indicate position of orthogonal views in XY plane. Scale bar, 10 μm. (TIFF) [file pntd.0006473.s002.tiff]

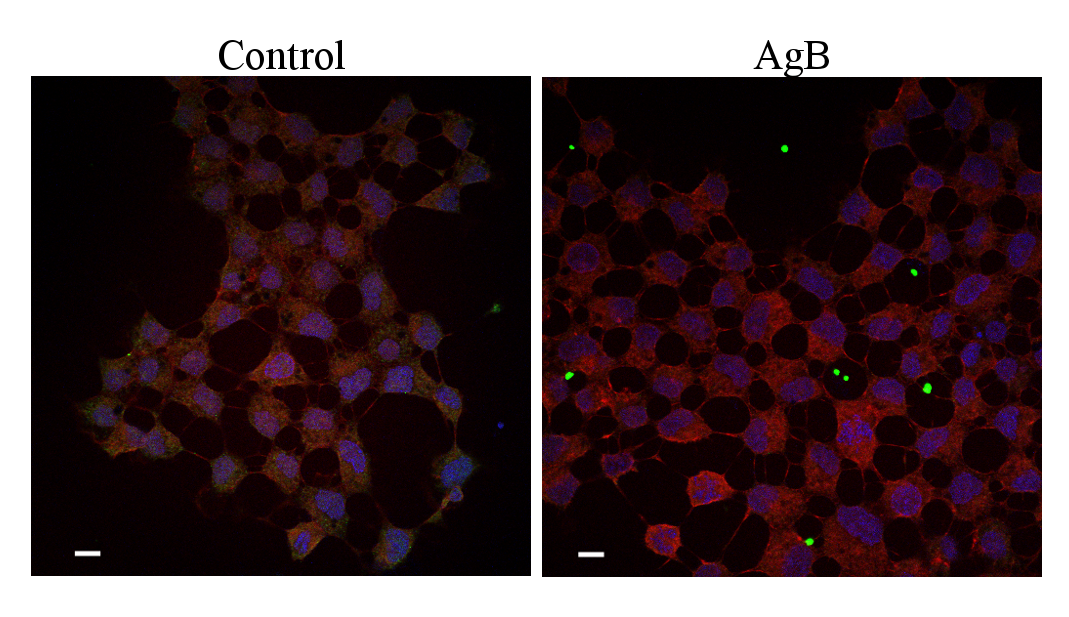

Supplement: S3 Fig — RH cells were exposed to AgB at 4°C for 4h, then fixed with paraformaldehyde. AgB was labelled with antibodies against subunits AgB8/1, 2 and 4 and an Alexa Fluor 488-conjugated secondary antibody (green). Nuclei and cytoskeleton were stained with DAPI (blue) and Alexa Fluor 594-conjugated phalloidin (red), respectively. Scale bar, 10 μm. (TIFF) [file pntd.0006473.s003.tiff]

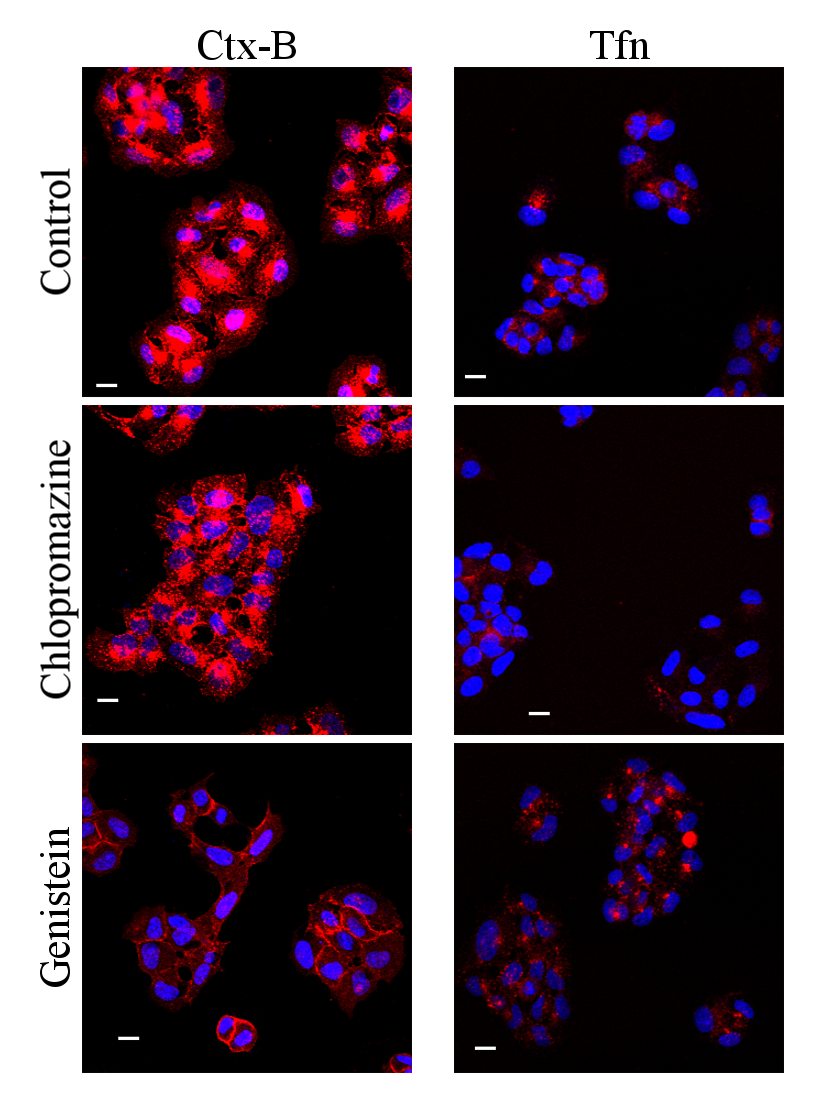

Supplement: S4 Fig — RH cells were treated with 100 μg/ml genistein or 5 μg/ml chlorpromazine 30 min prior to addition of Alexa Fluor 555-conjugated Ctx-B or Alexa Fluor 633-conjugated Tfn. Cells were fixed in 4% paraformaldehyde and nuclei were stained with DAPI (blue). Ctx-B and Tfn are shown in red. Scale bar 10 μm. (TIF) [file pntd.0006473.s004.tif]
